# A Nomogram Incorporating Sarcopenia and Nutritional Indicators for Mortality Prediction in HBV-Related Acute-Chronic Liver Failure

**DOI:** 10.3390/healthcare14040447

**Published:** 2026-02-11

**Authors:** Jiao Yuan, Wenting Peng, Chuan Jiang, Hui Liu, Shuo Wang, Ying Jiang, Bin Tan, Lei Fu, Shifang Peng

**Affiliations:** 1 Department of Infectious Diseases, Xiangya Hospital, Central South University, Changsha 410008, China; yuanjj2025@icloud.com (J.Y.); pengwenting_123@163.com (W.P.); jiangchuan1998@163.com (C.J.); 228102077@csu.edu.cn (Y.J.); tanbin0530@icloud.com (B.T.); fulei92@126.com (L.F.); 2 Department of Radiology, Xiangya Hospital, Central South University, Changsha 410008, China; 403799@csu.edu.cn; 3 Department of Pediatrics, The Second Xiangya Hospital, Central South University, Changsha 410011, China; wangshuo998@sina.com; 4 National Clinical Research Center for Geriatric Disorders, Xiangya Hospital, Central South University, Changsha 410008, China

**Keywords:** acute-on-chronic liver failure, hepatitis B virus, predictor, nutrient anabolism, nomogram

## Abstract

**Background**: The prognosis of acute-on-chronic liver failure (ACLF) is impaired by etiology heterogeneity across regions. Currently, prognostic models incorporating nutrient anabolism–related indicators for patients with hepatitis B virus (HBV)–associated ACLF are lacking. **Objectives**: This study aimed to construct a nomogram that incorporates nutrition-related indexes alongside traditional predictors to estimate 12-week mortality in HBV-ACLF. **Methods**: We retrospectively analyzed adult patients with HBV-ACLF treated at our department between May 2020 and December 2021. A total of 242 HBV-ACLF patients were enrolled and categorized into survivor (n = 174) and progression (n = 68) groups. Independent prognostic factors were identified using logistic regression analysis and incorporated into a nomogram. Nomogram performance was evaluated in terms of discrimination, calibration, and clinical utility, with internal validation using bootstrap resampling. **Results**: Patients in the progression group were older, more prone to hepatorenal syndrome and spontaneous peritonitis, and had lower levels of prothrombin activity, L3 skeletal muscle index and ceruloplasmin (all *p* < 0.05). These six independent predictors were incorporated into the nomogram, which demonstrated superior discrimination ability, with an area under the receiver operating characteristic curve of 0.95, enabling accurate identification of patients at high risk of short-term mortality. The Hosmer–Lemeshow test confirmed excellent calibration, decision curve analysis confirmed the clinical benefit, and bootstrap validation confirmed the robustness. **Conclusions**: The developed nomogram, incorporating nutritional status, may provide complementary information to support short-term risk stratification and clinical decision-making in patients with HBV-ACLF awaiting liver transplantation.

## 1. Introduction

Acute-on-chronic liver failure (ACLF) is a critical syndrome characterized by acute deterioration of liver function in chronic liver disease [1,2]. The etiology of ACLF varies substantially across geographic regions, with hepatitis B virus (HBV) infection remaining the predominant cause in China, accounting for approximately 70% of cases [3,4]. Although general management strategies, such as antiviral and hepatoprotective therapies, may help in mild cases, liver transplantation (LT) remains the only curative therapy for advanced ACLF [5]. Without timely LT, short-term mortality can reach 50–90% [6,7], underscoring the need for accurate risk stratification to optimize transplant prioritization.

Several predictive tools have been developed to guide organ allocation, including the Child–Pugh score [8], an early tool used to assess liver function severity, and the Model for End-Stage Liver Disease (MELD) and its modified version, MELD-sodium (MELD-Na). MELD, introduced by Malinchoc in 2000, incorporates international normalized ratio (INR), serum total bilirubin (TBIL), and serum creatinine to predict short-term mortality [9]. However, evidence suggests that some patients with advanced liver disease have low MELD scores despite high mortality risk [10]. Another study reported that MELD performance varies across the population [11]. Although MELD-Na was developed to improve prognostic accuracy [12], a large multicenter study demonstrated its limited ability to predict 90-day mortality in ACLF patients [13].

Given the substantial regional heterogeneity in ACLF etiology and clinical phenotype, population-specific models have been proposed. The Chinese Group on the Study of Severe Hepatitis B-ACLF (COSSH-ACLF) [14] and its simplified version, COSSH-ACLF II [15], were specifically developed for HBV-ACLF patients. The COSSH-ACLF II score integrates age, INR, TBIL, neutrophils, serum urea and hepatic encephalopathy [14]. However, due to their relatively short period of clinical application, further validation of these models remains necessary. Importantly, most existing prognostic systems largely overlook the role of nutrient anabolism in HBV-ACLF.

The liver plays a central role in nutrient synthesis and metabolic regulation. In end-stage liver disease, extensive hepatocellular damage leads to impaired nutrient anabolism, resulting in malnutrition [16]. Malnutrition, in turn, compromises hepatic regeneration and tissue repair, thereby accelerating hepatic functional deterioration [17,18]. It is highly prevalent among patients with cirrhosis and is related to poor survival, increased complications, and reduced resilience to physiological stressors such as surgery and infection among patients awaiting LT [19].

Impaired liver regeneration, a key determinant of ACLF outcome [20], depends not only on residual hepatic function but also on adequate metabolic substrates and preserved anabolic capacity [21]. Nutritional disturbances, such as sarcopenia and impaired hepatic protein synthesis, may critically limit regenerative potential and contribute to disease progression [22]. Nevertheless, the prognostic significance of nutritional status in HBV-ACLF has not been fully elucidated.

In this study, we developed a novel prognostic nomogram incorporating indicators of nutrient anabolism to improve short-term risk stratification and prognostic assessment in HBV-ACLF patients on the transplant waitlist. The performance of this model was further evaluated and compared with established prognostic systems, including MELD, MELD-Na and COSSH-ACLF II.

## 2. Methods

### 2.1. Study Population and Design

This was a single-center, retrospective study. The institutional ethics committee of Xiangya Hospital of Central South University approved the study (NO: 2025020255) and waived the requirement for obtaining informed consent from patients, because retrospective analysis did not affect patients’ privacy or clinical outcomes. The study was conducted in accordance with the ethical principles of the Declaration of Helsinki (1975).

The diagnosis of chronic hepatitis B was based on the Asian-Pacific clinical practice guidelines for the management of hepatitis [23]. ACLF was defined according to the Asian Pacific Association for the Study of the Liver definition (APASL) [24]. Given this study focused on HBV-associated ACLF in China, the diagnostic criteria proposed by COSSH were additionally applied to confirm patient eligibility [14]. Exclusion criteria included (a) hepatocellular carcinoma or malignancies in other organs; (b) age <18 years or pregnancy; (c) fungal or viral infections other than HBV; (d) liver diseases of non-HBV etiology (e.g., alcoholic, autoimmune, or hepatitis C-related); (e) preterminal chronic extrahepatic disease; (f) immunodeficiency disorders; and (g) incomplete clinical data.

From May 2020 to December 2021, 332 HBV-ACLF patients were admitted to our department. Of these, 90 were excluded, leaving 242 patients who were retrospectively analyzed. All study participants received standard medical treatments according to disease severity, including nucleos(t)ide analog–based antiviral therapy, bed rest, nutritional support, complication prevention, and artificial liver support when indicated.

**Figure 1:**
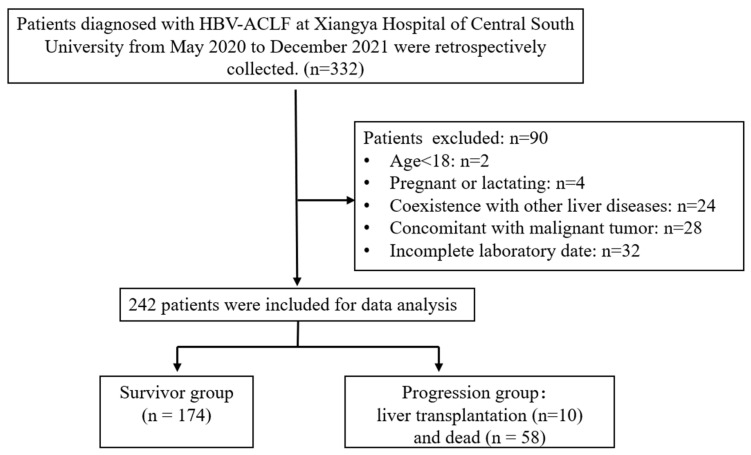
Flow chart of the patient inclusion process. Note: HBV hepatitis B virus, ACLF acute-on-chronic liver failure.

Patients were followed for 12 weeks through outpatient records or telephone interviews. According to clinical outcomes, patients who survived without transplantation within 12 w were categorized into the survivor group, whereas those who died or underwent liver transplantation were categorized into the progression group. The patient inclusion process is illustrated in Figure 1.

### 2.2. Data Collection

Demographic, laboratory, radiological, and clinical data were retrospectively extracted from the electronic medical record system. Demographic variables included age, sex and body mass index (BMI). Laboratory data included routine blood count, liver, kidney and thyroid function, coagulation parameters, pathogen detection, electrolyte and immunological assays, etc. Complete data on precipitating events were not available for all patients due to the retrospective design. However, the main precipitating events for HBV-ACLF in this study were stress, alcohol consumption, and discontinuation of nucleos(t)ide analog (NA) therapy.

Cirrhosis was estimated based on radiological evidence and clinical signs of portal hypertension. Hepatorenal syndrome (HRS) was diagnosed according to the International Club of Ascites Acute Kidney Injury (ICA AKI) criteria [25]. Spontaneous peritonitis (SP) was defined as an ascitic polymorphonuclear leukocyte count ≥ 250/mm^3^ in the absence of an intra-abdominal infection source [26]. Ascites was identified by imaging, paracentesis, or clinical signs. Gastrointestinal bleeding (GIB) was diagnosed based on the presence of hematemesis or melena. Hepatic encephalopathy (HE) was graded using the West Haven criteria [27].

Skeletal muscle mass at the third lumbar vertebral level (L3) was assessed using computed tomography or magnetic resonance imaging [28]. The cross-sectional skeletal muscle area at the L3 level was measured, including the erector spinae, psoas, transversus abdominis, interna oblique, external oblique, quadratus lumborum, and rectus abdominis. Skeletal muscle mass was quantified using Hounsfield unit thresholds ranging from −29 to +150 to minimize interference from ascites. The L3 skeletal muscle index (L3SMI) was calculated by normalizing the skeletal muscle area to the square of the patient’s height (m2).

Using this data, prognostic models including MELD, MELD-Na and COSSH-ACLF II were calculated. MELD was according to the standard formula [29]: MELD = 11.2 × ln (INR) + 9.6 × ln [creatinine (mg/dL)] + 3.8 × ln [TBIL (mg/dL)] + 6.4 (constant for liver disease etiology). MELD-Na score was calculated as follows: MELD-Na = MELD − Na − [0.025 × MELD × (140 − Na)] + 140. The COSSH-ACLF II score was calculated according to the published formula [13]: COSSH-ACLF II score= 1.649 × ln (INR) + 0.457 × HE score + 0.425 × ln (neutrophil count) + 0.396 × ln (TBIL) + 0.576 × ln (urea) + 0.033 × age.

### 2.3. Statistical Analysis

Quantitative variables were expressed as mean ± standard deviation (SD) or median (range) and compared using the Student’s *t*-test or the Mann–Whitney U-test, as appropriate. Categorical variables were expressed as counts (percentage) and compared using the χ^2^ test.

The primary endpoint was 12-week mortality. Variables associated with this endpoint were first analyzed using univariate logistic regression. Variables with *p* < 0.05 were subsequently entered into multivariable logistic regression analysis to identify independent predictors. To assess the stability of predictors, two models were constructed. Model I adjusted for demographic factors, age, sex and BMI; Model II adjusted for age, sex, BMI, L3SMI, HRS, SP, HE, TBIL, direct bilirubin (DBIL), estimated glomerular filtration rate (eGFR), prothrombin time (PT), INR, prothrombin activity (PTA), ceruloplasmin (CER), complement 3 (C3) and immunoglobulin G (IgG).

The identified independent predictors were imported into a predictive nomogram using R software. Discriminative performance was evaluated using receiver operating characteristic (ROC) curve analysis, and areas under the curves (AUCs) were compared between models using the Z test [30]. Calibration and clinical benefit were assessed using the Hosmer–Lemeshow (H-L) goodness-of-fit test and decision curve analysis (DCA), respectively.

Internal validation was performed using bootstrap resampling with 1000 iterations to assess model stability and optimism. All statistical analyses were conducted using IBM SPSS Statistics (version 25.0) and R software (version 4.0.5). A two-sided *p* value < 0.05 was considered statistically significant.

## 3. Results

### 3.1. General Characteristics of Patients

As shown in Table 1, a total of 242 patients diagnosed with HBV-ACLF were included in the analysis. Among them, 208 (85.95%) were male, with a median age of 47.26 years, and 136 (56.20%) had existing cirrhosis. All eligible patients received NA therapy during hospitalization. Before ACLF onset, 226 patients (93.4%) were receiving continuous NA therapy, 5 patients (2.1%) initiated NA therapy de novo at admission, and 11 patients (4.5%) had discontinued NA therapy before ACLF onset (Supplementary Table S1). With respect to specific medications, 140 patients (57.85%) were treated with entecavir (ETV), while 102 (42.15%) received either tenofovir disoproxil fumarate (TDF) or tenofovir alafenamide (TAF). According to clinical outcomes, 174 patients (71.90%) were assigned to the survivor group and 68 patients (28.10%) to the progression group.

Compared with survivors, patients in the progression group exhibited a significantly higher prevalence of major complications, including HRS (17.65% vs. 2.87%), SP (86.76% vs. 56.90%) and HE (23.53% vs. 5.17%) (*p* < 0.001 for all). In addition, patients in the progression group were significantly older (*p* < 0.001) and showed more severe biochemical abnormalities, characterized by higher levels of TBIL, DBIL, PT, INR and IgG (all *p* < 0.05). Conversely, the progression group demonstrated significantly lower levels of L3SMI, eGFR, PTA, CER and C3 (all *p* < 0.01).

### 3.2. Univariate and Multivariate Analysis Screening Risk Factors

Univariate analysis (Table 2) identified age, TBIL, DBIL, PT, INR, IgG and the presence of HRS, SP and HE as potential risk factors for poor clinical course in HBV-ACLF patients (all *p* < 0.05). In contrast, higher levels of L3SMI, hemoglobin, eGFR, Na, PTA, CER and FT3 were associated with a more favorable prognosis (all *p* < 0.05). To identify factors independently associated with prognosis, variables that were significant in the univariate analysis were entered into multivariate logistic regression models. Two models were constructed with stepwise inclusion of covariates to assess the stability of the identified predictors.

The results showed that age (OR 1.08, *p* = 0.0062), HRS (OR 29.76, *p* = 0.0033) and SP (OR 10.24, *p* = 0.0030) were positively associated with poor prognosis. In contrast, L3SMI (OR 0.80, *p* < 0.0001), PTA (OR 0.92, *p* = 0.0099) and CER (OR 0.99, *p* = 0.0164) were negatively associated with disease progression. These relationships remained consistent and stable across both multivariable models (Table 3). Variables that did not reach statistical significance in the multivariate analysis are presented in the Supplemental Table S2.

### 3.3. Predictive Model Construction for HBV-ACLF

**Figure 2:**
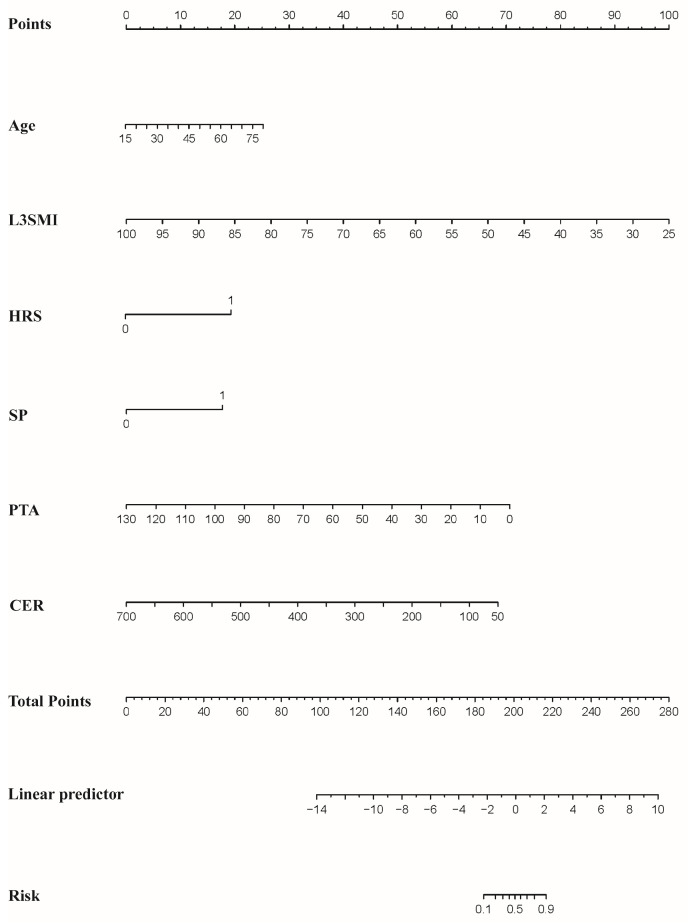
Nomogram for predicting 12-week mortality of HBV-ACLF patients. Note: L3SMI L3 skeletal muscle index, HRS hepatorenal syndrome, SP spontaneous peritonitis, PTA prothrombin activity, CER ceruloplasmin.

A nomogram was established using the six independent predictors identified in multivariable analysis (Figure 2). To use the nomogram, the value of each variable is located on its corresponding axis, and a vertical line is drawn upward to the “Points” scale to determine the score for that variable. The individual scores are then summed to obtain a total score. Finally, this total score is projected downward to the probability axis to estimate the predicted 12-week mortality risk for each patient with HBV-ACLF. This model, combining age, L3SMI, HRS, SP, PTA and CER, was named ALHSPC.

### 3.4. Discrimination, Calibration and Clinical Benefit

**Figure 3:**
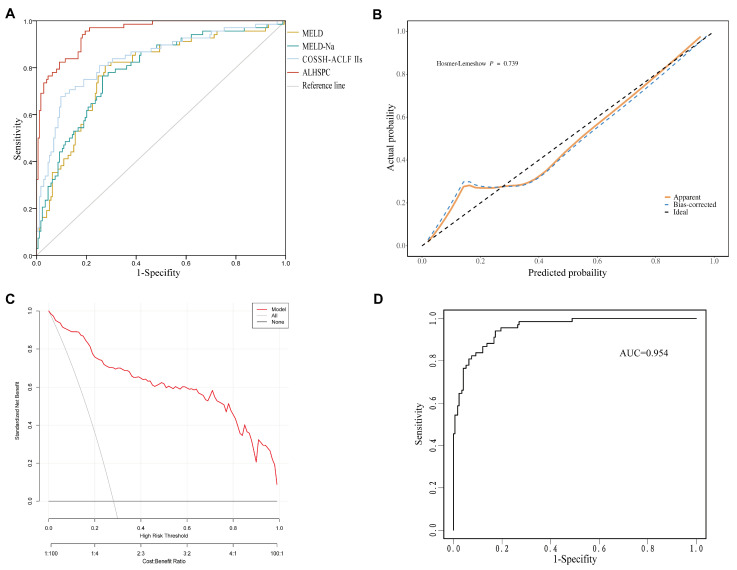
Performance of the ALHSPC model in patients with HBV-ACLF. (**A**) ROC comparing the discriminative ability of the ALHSPC with MELD, MELD-Na, and COSSH-ACLF IIs. (**B**) Calibration curve showing the agreement of the ALHSPC model. (**C**) Decision curve analysis showing the clinical utility of the ALHSPC model. (**D**) Bootstrap internal validation demonstrating the stability of the ALHSPC model. Note: MELD the Model For End-Stage Liver Disease, MELD-Na MELD-sodium, COSSH-ACLF IIs Chinese Severe Hepatitis B Study Group- acute-on-chronic liver failure II score, ALHSPC a new developed model combined of age, L3 skeletal muscle index, hepatorenal syndrome, spontaneous peritonitis, prothrombin activity and ceruloplasmin.

We compared the discriminative accuracy of ALHSPC with other prognostic models by AUC (Figure 3A). The ALHSPC model achieved an AUC of 0.95 (95%CI 0.92–0.97), which was significantly higher than that of MELD (0.77, 95%CI 0.70–0.84), MELD-Na (0.78, 95%CI 0.72–0.85), and COSSH-ACLF IIs (0.83, 95%CI 0.76–0.88) (all *p* < 0.001).

Using an optimal cut-off value of 0.288, the ALHSPC model yielded an accuracy of 0.85, sensitivity of 0.96, specificity of 0.80, PPV of 0.66, and NPV of 0.98. Overall, these performance metrics were superior to those of the MELD and MELD-Na models but were slightly inferior to COSSH-ACLF IIs on specificity and PPV (Table 4).

Model calibration was assessed using the H-L goodness-of-fit test, which yielded a χ^2^ value of 5.17 (*p* = 0.739), indicating an excellent fit between the predicted and observed outcomes (Figure 3B). DCA further demonstrated the clinical benefit of the ALHSPC model, showing a higher net benefit across a wide range of threshold probabilities compared with the strategies of treating all or treating none (Figure 3C).

Internal validation using bootstrap resampling confirmed the robustness of the model. The bootstrap analysis showed stable discriminative performance with minimal optimism, supporting the reliability and internal stability of the ALHSPC model (Figure 3D).

## 4. Discussion

HBV-ACLF is among the most common indications for LT. The etiology, clinical characteristics and outcomes of ACLF vary substantially across geographic regions and populations, leading to marked differences in diagnosis, treatment and prognosis [31]. In Eastern regions, HBV remains the predominant cause of ACLF, whereas alcohol-related liver disease is more prevalent in Western countries. Regarding complications, liver and clotting failure are more frequently observed in HBV-ACLF, while brain and kidney failure are more common in non-HBV-ACLF. Additionally, the 28- and 90-day survival rates are significantly lower in HBV-ACLF patients compared with non-HBV-ACLF [14,32,33].

These regional variations suggest that widely used prognostic models such as MELD and MELD-Na may be suboptimal for predicting ACLF progression in Eastern populations, as they were largely derived from Western cohorts in which alcoholism and non-alcoholic steatohepatitis predominate. Although the COSSH-ACLF and COSSH-ACLF II models were developed specifically for HBV-ACLF, their effectiveness still requires further validation [13,14]. Therefore, there remains a pressing need for a dedicated prognostic tool tailored to HBV-ACLF to assist clinicians in disease severity assessment and resource allocation.

In this study, we focused exclusively on HBV-related ACLF and enrolled patients according to Asian-Pacific criteria, which are widely used in China. Our analyses yielded three major findings: (1) We constructed the ALHSPC model incorporating indicators of nutrient anabolism to evaluate outcomes in HBV-ACLF patients, which showed superior discriminative and predictive performance. (2) L3SMI and CER were identified as independent prognostic factors and were integrated with established clinical parameters to predict 12-week survival. (3) Because HBV infection was the sole etiology in our cohort, the ALHSPC model may be particularly applicable to Asian populations with similar disease characteristics. Nevertheless, its nutritional–anabolic framework may allow future adaptation and validation in ACLF populations with different etiologies.

Notably, the ALHSPC nomogram is not intended to replace existing liver transplant prioritization systems such as MELD or COSSH-ACLF II. Rather, it may serve as a complementary tool to refine short-term risk stratification, particularly among patients with similar conventional scores but heterogeneous nutritional and anabolic status.

In our model, six variables—age, L3SMI, HRS, SP, PTA and CER—were identified as independent risk factors for 12-week progression in HBV-ACLF patients. In line with the COSSH-ACLF and COSSH-ACLF II scoring systems, age emerged as a significant independent predictor, reflecting the regenerative capacity of hepatic parenchymal cells [13,14]. HRS and SP are common complications of ACLF, and the presence of either condition has been strongly associated with adverse outcomes [34,35]. Consistent with previous studies, our findings confirmed that HBV-ACLF patients complicated by HRS or SP experienced worse clinical outcomes and higher 12-week mortality, highlighting their importance as prognostic indicators.

PTA is a diagnostic criterion for ACLF in both the APASL and Chinese liver failure guidelines. It is a sensitive indicator of hepatocyte necrosis, coagulation failure, and hepatic synthetic capacity [24,36]. Unlike most existing prognostic models that incorporate the INR, our study identified PTA as an independent predictive indicator of HBV-ACLF progression, underscoring its clinical relevance in this population.

Sarcopenia, assessed using the L3SMI, has been recognized as an independent predictor of pre-transplant mortality and adverse post-transplant outcomes in patients with liver cirrhosis [37,38]. In addition, unlike traditional prognostic variables such as sex or age, sarcopenia is potentially modifiable through nutritional support and physical intervention [39]. In our cohort, L3SMI was significantly lower in individuals with disease progression, indicating its independent prognostic value in HBV-ACLF.

CER is a hepatically synthesized copper-containing glycoprotein accounting for more than 90% of circulating copper [40]. Reduced CER levels reflect impaired hepatic synthetic and metabolic capacity and may disrupt multiple anabolic processes, including trace metal homeostasis and oxidative metabolism [41]. Although CER has been implicated in various chronic liver diseases, its prognostic significance in ACLF has remained unclear [42]. Our findings demonstrate that low CER levels are associated with worse outcomes in HBV-ACLF, supporting its role as an independent predictor.

Notably, L3SMI and ceruloplasmin capture complementary aspects of impaired nutrient anabolism in HBV-ACLF. L3SMI reflects extrahepatic protein and amino acid reserves available to meet the increased metabolic demands during acute decompensation, whereas CER represents intrahepatic synthetic capacity under stress conditions. Together, these abnormalities characterize an unfavorable anabolic state that may limit the liver’s ability to sustain recovery during acute injury, thereby contributing to disease progression and poor prognosis.

There were several limitations in the present study. First, our model was developed based on a single-center cohort with a relatively small sample size, and external validation in independent cohorts was not performed, which may limit the generalizability of the findings. Therefore, validation in larger, multicenter HBV-ACLF cohorts is warranted. Second, the study population was restricted to HBV-related ACLF, limiting applicability to Western populations where alcohol-related liver disease and non-alcoholic steatohepatitis predominate. Third, as a retrospective study, we were unable to quantify nutritional intake, refine nutritional assessment scales, or dynamically monitor predictive indicators during hospitalization.

## 5. Conclusions

In conclusion, this study highlights the crucial role of nutrient anabolism in predicting 12-week mortality in HBV-ACLF patients. Nutritional anabolic disorders may provide valuable complementary information alongside traditional predictors, supporting short-term risk stratification and transplant-related decision-making. The ALHSPC model demonstrated favorable performance in predicting disease severity in HBV-ACLF, with superior accuracy, discrimination, and calibration compared to other models, and showed good robustness in internal validation.

## Figures and Tables

**Table 1:** Baseline features between the survivor and progression groups.

Variables	Survivor (n = 174)	Progression (n = 68)	Standardize Diff.	*p*-Value
Age (years)	44.84 ± 12.03	53.46 ± 11.48	0.73 (0.44, 1.02)	<0.001
BMI (kg/m^2^)	23.94 ± 3.75	23.17 ± 3.85	0.20 (−0.08, 0.48)	0.154
L3SMI (cm^2^/m^2^)	57.78 ± 8.90	46.25 ± 8.89	1.30 (0.99, 1.60)	<0.001
Neutrophil (×10^9^/L)	4.27 ± 2.23	4.75 ± 2.38	0.21 (−0.07, 0.49)	0.138
WBC (×10^9^/L)	6.36 ± 3.89	6.53 ± 2.74	0.05 (−0.23, 0.33)	0.736
Hemoglobin (g/L)	138.48 ± 130.84	116.02 ± 24.89	0.24 (−0.04, 0.52)	0.162
Albumin (g/L)	30.45 ± 4.70	29.41 ± 4.02	0.24 (−0.04, 0.52)	0.108
Globulin (g/L)	29.25 ± 6.91	30.06 ± 8.08	0.11 (−0.17, 0.39)	0.439
TBIL (μmol/L)	289.43 ± 140.29	407.40 ± 157.27	0.79 (0.50, 1.08)	<0.001
DBIL (μmol/L)	172.04 ± 83.59	223.05 ± 86.53	0.60 (0.31, 0.88)	<0.001
ALT (U/L)	495.24 ± 552.34	382.98 ± 377.67	0.24 (−0.05, 0.52)	0.127
AST(U/L)	383.64 ± 469.21	389.46 ± 459.45	0.01 (−0.27, 0.29)	0.931
TBA (μmol/L)	214.61 ± 92.94	236.73 ± 73.40	0.26 (−0.02, 0.55)	0.08
Urea (mmol/L)	4.88 ± 2.95	5.68 ± 3.49	0.24 (−0.04, 0.53)	0.076
Creatinine (μmol/L)	90.76 ± 67.68	96.10 ± 40.62	0.10 (−0.18, 0.38)	0.543
eGFR (mL/min/1.73 m^2^)	90.71 ± 21.24	81.42 ± 23.46	0.42 (0.13, 0.70)	0.003
Uric acid (μmol/L)	209.61 ± 248.73	170.90 ± 99.49	0.20 (−0.08, 0.49)	0.215
Na (mmol/L)	144.97 ± 96.84	136.04 ± 4.25	0.13 (−0.15, 0.41)	0.448
K (mmol/L)	3.77 ± 0.48	3.79 ± 0.53	0.02 (−0.26, 0.30)	0.883
Ca (mmol/L)	2.13 ± 0.14	2.15 ± 0.13	0.09 (−0.19, 0.37)	0.518
PT (sec)	19.61 ± 5.63	26.69 ± 11.94	0.76 (0.47, 1.05)	<0.001
INR	1.78 ± 0.92	5.53 ± 24.13	0.22 (−0.06, 0.50)	0.042
PTA (%)	48.39 ± 18.47	31.72 ± 11.94	1.07 (0.78, 1.37)	<0.001
CER (mg/L)	234.51 ± 81.42	180.10 ± 53.41	0.79 (0.50, 1.08)	<0.001
C3 (mg/L)	476.94 ± 195.74	370.02 ± 177.62	0.57 (0.29, 0.86)	<0.001
C4 (mg/L)	122.35 ± 56.64	113.51 ± 74.01	0.13 (−0.15, 0.41)	0.32
IgG (g/L)	19.41 ± 5.32	21.13 ± 7.04	0.28 (−0.00, 0.56)	0.04
FT3 (pmol/L)	3.35 ± 2.82	2.67 ± 1.17	0.32 (0.03, 0.60)	0.055
FT4 (pmol/L)	18.66 ± 11.21	18.45 ± 10.77	0.02 (−0.26, 0.30)	0.896
TSH (mIU/L)	1.88 ± 3.21	1.67 ± 2.85	0.07 (−0.21, 0.35)	0.642
HBVDNA (lg IU/mL)	4.63 ± 1.81	4.46 ± 1.75	0.10 (−0.18, 0.38)	0.496
HBsAg (lg IU/mL)	3.14 ± 1.07	3.08 ± 1.35	0.05 (−0.23, 0.33)	0.688
HBeAg positive	61 (35.06%)	14 (20.59%)	0.33 (0.05, 0.61)	0.051
Cirrhosis	94 (54.02%)	42 (61.76%)	0.16 (−0.12, 0.44)	0.275
NA type				
TDF/TAF	76 (43.68%)	26 (38.24%)	0.11 (−0.17, 0.39)	0.441
ETV	98 (56.32%)	42 (61.76%)		
Complications (no.)				
HRS	5 (2.87%)	12 (17.65%)	0.50 (0.22, 0.79)	<0.001
SP	99 (56.90%)	59 (86.76%)	0.50 (0.22, 0.79)	<0.001
Ascites	81 (46.55%)	41 (60.29%)	0.28 (−0.00, 0.56)	0.055
GIB	4 (2.30%)	2 (2.94%)	0.04 (−0.24, 0.32)	0.773
HE	9 (5.17%)	16 (23.53%)	0.54 (0.26, 0.83)	<0.001
Male (no.)	152 (87.36%)	56 (82.35%)	0.14 (−0.14, 0.42)	0.314

Note: BMI body mass index, L3SMI L3 skeletal muscle index, WBC white blood cell, TBIL total bilirubin, DBIL direct bilirubin, ALT alanine aminotransferase, AST aspartate aminotransferase, TBA total bile acid, eGFR estimated glomerular filtration rate, PT prothrombin time, INR international normalized ratio, PTA prothrombin activity, CER ceruloplasmin, C3 complement 3, C4 complement 4, IgG immunoglobulin G, FT3 free triiodothyronine, FT4 free thyroxine, TSH thyroid Stimulating Hormone, HBV hepatitis B virus DNA, HBsAg Hepatitis B surface antigen, HBeAg, hepatitis B e antigen, NA nucleos(t)ide analogue, ETV entecavir, TDF tenofovir disoproxil fumarate, TAF tenofovir alafenamide, HRS hepatorenal syndrome, SP spontaneous peritonitis, GIB gastrointestinal bleeding, HE hepatic encephalopathy.

**Table 2:** Univariate logistic regression analysis on prognosis of HBV-ACLF patients.

Variables	Statistics	OR (95%CI)	*p*-Value
Age (years)	47.26 ± 12.48	1.06 (1.03, 1.09)	<0.0001
BMI (kg/m^2^)	23.72 ± 3.79	0.95 (0.87, 1.02)	0.1542
L3SMI (cm^2^/m^2^)	54.54 ± 10.29	0.86 (0.82, 0.90)	<0.0001
Neutrophil (×10^9^/L)	4.41 ± 2.28	1.09 (0.97, 1.23)	0.1412
WBC (×10^9^/L)	6.41 ± 3.60	1.01 (0.94, 1.09)	0.7362
Hemoglobin (g/L)	132.17 ± 112.08	0.98 (0.97, 1.00)	0.0103
Albumin (g/L)	30.16 ± 4.53	0.95 (0.89, 1.01)	0.109
Globulin (g/L)	29.48 ± 7.25	1.02 (0.98, 1.06)	0.438
TBIL (μmol/L)	322.58 ± 154.36	1.01 (1.00, 1.01)	<0.0001
DBIL (μmol/L)	186.37 ± 87.32	1.01 (1.00, 1.01)	<0.0001
ALT (U/L)	464.03 ± 511.54	1.00 (1.00, 1.00)	0.1301
AST (U/L)	385.28 ± 465.55	1.00 (1.00, 1.00)	0.9302
TBA (μmol/L)	220.82 ± 88.30	1.00 (1.00, 1.01)	0.0811
Urea (mmol/L)	5.11 ± 3.13	1.08 (0.99, 1.17)	0.0881
Creatinine (μmol/L)	92.26 ± 61.26	1.00 (1.00, 1.01)	0.5561
eGFR (ml/min/1.73 m^2^)	88.10 ± 22.23	0.98 (0.97, 0.99)	0.0042
Uric acid (μmol/L)	198.73 ± 217.86	1.00 (0.99, 1.00)	0.1301
Na (mmol/L)	142.46 ± 82.17	0.89 (0.82, 0.96)	0.0036
K (mmol/L)	3.78 ± 0.49	1.04 (0.59, 1.85)	0.8828
Ca (mmol/L)	2.14 ± 0.13	2.00 (0.25, 16.31)	0.5164
PT (sec)	21.60 ± 8.52	1.14 (1.08, 1.19)	<0.0001
INR	2.83 ± 12.86	2.14 (1.40, 3.27)	0.0004
PTA (%)	43.71 ± 18.46	0.91 (0.89, 0.94)	<0.0001
CER (mg/L)	219.22 ± 78.44	0.99 (0.98, 0.99)	<0.0001
C3 (mg/L)	446.90 ± 196.46	1.00 (0.99, 1.00)	0.0002
C4 (mg/L)	119.87 ± 61.98	1.00 (0.99, 1.00)	0.3202
IgG (g/L)	19.89 ± 5.89	1.05 (1.00, 1.10)	0.043
FT3 (pmol/L)	3.16 ± 2.49	0.61 (0.42, 0.88)	0.0094
FT4 (pmol/L)	18.60 ± 11.07	1.00 (0.97, 1.02)	0.8953
TSH (mIU/L)	1.82 ± 3.11	0.98 (0.88, 1.08)	0.6417
HBVDNA (lg IU/mL)	4.58 ± 1.79	0.95 (0.81, 1.11)	0.494
HBsAg (lg IU/mL)	3.13 ± 1.15	0.95 (0.75, 1.21)	0.6863
HBeAg positive	75 (30.99%)	0.48 (0.25, 0.93)	0.0507
Cirrhosis	136 (56.20%)	1.37 (0.78, 2.44)	0.2761
NA type			
TDF/TAF	102 (42.15%)	0.80 (0.45, 1.42)	0.4413
ETV	140 (57.85%)		
Complications (no.)			
HRS	17 (7.02%)	7.24 (2.44, 21.46)	0.0004
SP	158 (65.29%)	4.97 (2.32, 10.65)	<0.0001
Ascites	122 (50.41%)	1.74 (0.99, 3.08)	0.0559
GIB	6 (2.48%)	1.29 (0.23, 7.20)	0.7733
HE	25 (10.33%)	5.64 (2.35, 13.52)	0.0001
Male (no.)	208 (85.95%)	1.48 (0.69, 3.19)	0.3161

Note: BMI body mass index, L3SMI L3 skeletal muscle index, WBC white blood cell, TBIL total bilirubin, DBIL direct bilirubin, ALT alanine aminotransferase, AST aspartate aminotransferase, TBA total bile acid, eGFR estimated glomerular filtration rate, PT prothrombin time, INR international normalized ratio, PTA prothrombin activity, CER ceruloplasmin, C3 complement 3, C4 complement 4, IgG immunoglobulin G, FT3 free triiodothyronine, FT4 free thyroxine, TSH thyroid Stimulating Hormone, HBV hepatitis B virus DNA, HBsAg Hepatitis B surface antigen, HBeAg, hepatitis B e antigen, NA nucleos(t)ide analogue, ETV entecavir, TDF tenofovir disoproxil fumarate, TAF tenofovir alafenamide, HRS hepatorenal syndrome, SP spontaneous peritonitis, GIB gastrointestinal bleeding, HE hepatic encephalopathy.

**Table 3:** Multivariate logistic regression on prognosis of HBV-ACLF patients.

	Adjust I		Adjust II	
	OR (95%CI)	*p*-Value	OR (95%CI)	*p*-Value
Age	1.06 (1.03, 1.09)	<0.0001	1.08 (1.02, 1.14)	0.0062
L3SMI	0.81 (0.76, 0.86)	<0.0001	0.80 (0.73, 0.87)	<0.0001
HRS	5.92 (1.88, 18.65)	0.0024	29.76 (3.09, 286.68)	0.0033
SP	5.88 (2.56, 13.53)	<0.0001	10.24 (2.20, 47.64)	0.0030
PTA	0.91 (0.88, 0.94)	<0.0001	0.92 (0.87, 0.98)	0.0099
CER	0.98 (0.98, 0.99)	<0.0001	0.99 (0.97, 1.00)	0.0164

Result variable: prognosis. Exposure variable: age, L3SMI, HRS, SP, PTA, CER. Adjust I model in age adjusted for: sex, BMI. Adjust I model in L3SMI, HRS, SP, PTA, CER adjusted for age, sex, BMI. Adjust II model in age, L3SMI, HRS, SP, PTA, CER adjusted for age, sex, BMI, L3SMI, HRS, SP, HE, TBIL, DBIL, eGFR, PT, INR, PTA, CER, C3 and IgG, except for the variable being analyzed itself. Note: BMI body mass index, L3SMI L3 skeletal muscle index, HRS hepatorenal syndrome, SP spontaneous peritonitis, PTA prothrombin activity, CER ceruloplasmin, HE hepatic encephalopathy, TBIL total bilirubin, DBIL direct bilirubin, eGFR estimated glomerular filtration rate, PT prothrombin time, INR international normalized ratio, C3 complement 3, IgG immunoglobulin G.

**Table 4:** Comparison of the predictive performance of ALHSPC and other prognostic models in HBV-ACLF patients.

Model	AUC (95%CI)	Accuracy	Sensitivity	Specificity	PPV	NPV	Cut Off	*p*-Value
ALHSPC	0.95 (0.92–0.97)	0.85	0.96	0.80	0.66	0.98	−1.72	
MELD	0.77 (0.70–0.84)	0.75	0.81	0.72	0.53	0.91	24.59	<0.001
MELD-Na	0.78 (0.72–0.85)	0.74	0.76	0.74	0.53	0.89	25.87	<0.001
COSSH-ACLF IIs	0.83 (0.76–0.88)	0.84	0.68	0.90	0.73	0.88	6.94	<0.001

*p*-value, AUC of ABILI vs. AUC of other predictive models. Note: MELD the Model For End-Stage Liver Disease, MELD-Na MELD-sodium, COSSH-ACLF IIs Chinese Severe Hepatitis B Study Group- acute-on-chronic liver failure II score, ALHSPC a new developed model combined of age, L3 skeletal muscle index, hepatorenal syndrome, spontaneous peritonitis, prothrombin activity and ceruloplasmin, AUC area under the receiver operating characteristic curve, PPV positive predictive value, NPV negative predictive value.

## Data Availability

The data presented in this study are available on request from the corresponding author due to ethical regulations.

## Supplementary Materials

The following supporting information can be downloaded at: https://www.mdpi.com/article/10.3390/healthcare14040447/s1, Table S1. Timing of NA therapy in patients with HBV-ACLF. Table S2 Multivariate logistic regression for prognosis of HBV-ACLF.
