# Supplementary material for: A Nomogram Incorporating Sarcopenia and Nutritional Indicators for Mortality Prediction in HBV-Related Acute-Chronic Liver Failure
Source: Healthcare (Basel). 2026 Feb 11;14(4):447. doi: 10.3390/healthcare14040447 (PMC12940219; doi:10.3390/healthcare14040447)
Supplement: Supplementary file 1 [file healthcare-14-00447-s001.zip › healthcare-4061513-supplementary.pdf]

Supplementary Table S1. Timing of NA therapy in patients with HBV-ACLF

**NA therapy timing**	**n (%)**
Continuous NA therapy before ACLF onset	226 (93.4)
De novo NA initiation at hospital admission	5 (2.1)
NA discontinuation before ACLF onset	11 (4.5)
Total	242 (100.0)

Note: NA nucleos(t)ide analogue

Supplemental Table S2 Multivariate logistic regression for prognosis of HBV-ACLF

	**Adjust I**		**Adjust II**	
	**OR (95%CI)**	**P-value**	**OR (95%CI)**	**P-value**
HE	5.81 (2.29, 14.73)	0.0002	1.75 (0.36, 8.49)	0.4861
TBIL	1.01 (1.00, 1.01)	<0.0001	1.01 (1.00, 1.02)	0.1995
DBIL	1.01 (1.00, 1.01)	<0.0001	0.99 (0.97, 1.01)	0.4567
eGFR	0.99 (0.98, 1.00)	0.1045	1.00 (0.97, 1.03)	0.9001
PT	1.14 (1.08, 1.20)	<0.0001	0.94 (0.83, 1.06)	0.2893
INR	0.98 (0.98, 0.99)	0.0017	1.56 (0.78, 3.13)	0.2118
C3	1.00 (0.99, 1.00)	0.0004	1.00 (1.00, 1.00)	0.5445
IgG	1.04 (0.99, 1.09)	0.1370	1.05 (0.96, 1.15)	0.2872

Result variable: prognosis. Exposure variable: HE, TBIL, DBIL, eGFR, PT, INR, C3 and Ig G. Adjust I model adjusted for age, sex, BMI. Adjust II model in HE, TBIL, DBIL, eGFR, PT, INR, C3 and Ig G adjusted for age, sex, BMI, L3SMI, HRS, SP, HE, TBIL, DBIL, eGFR, PT, INR, PTA, CER, C3 and Ig G, except for the variable being analyzed itself. Note: BMI body mass index, L3SMI L3 skeletal muscle index, HRS hepatorenal syndrome, SP spontaneous peritonitis, PTA prothrombin activity, CER ceruloplasmin, BMI body mass index, HE hepatic encephalopathy, TBIL total bilirubin, DBIL direct bilirubin, eGFR estimated glomerular filtration rate, PT prothrombin time, INR international normalized ratio, C3 complement 3, IgG immunoglobulin G.
